# A computational multiscale agent-based model for simulating spatio-temporal tumour immune response to PD1 and PDL1 inhibition

**DOI:** 10.1098/rsif.2017.0320

**Published:** 2017-09-20

**Authors:** Chang Gong, Oleg Milberg, Bing Wang, Paolo Vicini, Rajesh Narwal, Lorin Roskos, Aleksander S. Popel

**Affiliations:** 1 Department of Biomedical Engineering, Johns Hopkins University School of Medicine, Baltimore, MD 21205, USA; 2 Department of Oncology, Sidney Kimmel Comprehensive Cancer Center, Johns Hopkins University School of Medicine, Baltimore, MD 21205, USA; 3 MedImmune, Mountain View, CA, USA; 4 MedImmune, Cambridge, UK; 5 MedImmune, Gaithersburg, MD, USA

**Keywords:** immuno-oncology, immunotherapy, immune checkpoint, systems biology, biomarker

## Abstract

When the immune system responds to tumour development, patterns of immune infiltrates emerge, highlighted by the expression of immune checkpoint-related molecules such as PDL1 on the surface of cancer cells. Such spatial heterogeneity carries information on intrinsic characteristics of the tumour lesion for individual patients, and thus is a potential source for biomarkers for anti-tumour therapeutics. We developed a systems biology multiscale agent-based model to capture the interactions between immune cells and cancer cells, and analysed the emergent global behaviour during tumour development and immunotherapy. Using this model, we are able to reproduce temporal dynamics of cytotoxic T cells and cancer cells during tumour progression, as well as three-dimensional spatial distributions of these cells. By varying the characteristics of the neoantigen profile of individual patients, such as mutational burden and antigen strength, a spectrum of pretreatment spatial patterns of PDL1 expression is generated in our simulations, resembling immuno-architectures obtained via immunohistochemistry from patient biopsies. By correlating these spatial characteristics with *in silico* treatment results using immune checkpoint inhibitors, the model provides a framework for use to predict treatment/biomarker combinations in different cancer types based on cancer-specific experimental data.

## 1. Introduction

The immune system has been hypothesized to play an active role in detecting constantly arising nascent tumours from normal tissues and preventing cancer development. This hypothesis, later coined as immunosurveillance [1], is substantiated by experimental evidence, including the identification of tumour associated antigens [2]. Our current understanding of immunosurveillance consists of three major phases: elimination, equilibrium and escape [3]. In the elimination phase, the immune system detects immunogenic tumours via mutational or abnormally expressed genes and mounts an adaptive immune response by mobilizing actors such as cytotoxic T lymphocytes in an attempt to kill the nascent transformed tumour cells [4]. However, incomplete elimination leads to survival of some tumour cells, which eventually acquire features that can help them evade the immune detection and ultimately results in tumour escape. One important route towards such escape is created as tumour cells hijack the regulatory pathways of the immune system to suppress its functionality.

The programmed cell death protein-1 (PD1) is one of those key factors [5]. Normally in an immune response, PD1 and its ligand PDL1 function as an immune checkpoint pathway to maintain tolerance to ‘self’ material and prevent excessive immune activities. Nonetheless, under protracted immune stress, PDL1 expression can be induced on cancer cells and other cells in the tumour microenvironment (TME), suppressing the host's anti-tumour immunity in many cancers such as melanoma and non-small cell lung cancer (NSCLC). Recent research into immuno-oncology has been focusing on modulating those regulatory factors of the immune system in hope of unleashing the natural power of anti-tumour immune response to treat a wide spectrum of cancers with less adverse effects and long-lasting memory. Immune checkpoint inhibitors, a new class of therapeutic agents, have been developed or are currently under development based on this principle [6]. These are typically monoclonal antibodies specifically targeting checkpoint proteins including PD1, PDL1, cytotoxic T-lymphocyte-associated protein 4 and many others. In clinical trials, therapies employing these agents have exhibited durable responses and improved survival of patients across a range of cancers including advanced melanoma, NSCLC and bladder cancer, and have thus been granted regulatory approval from the Food and Drug Administration for those indications [7–9].

However, unaddressed issues remain that limit a wider and more effective application of immunotherapies. First, although patients who respond to anti-PD1 showed significant tumour reduction and improved progression-free survival, the response rate is relatively low, e.g. about 30–40% in advanced melanoma and 20% in NSCLC [10]. In order to identify patients that will most likely benefit from such therapies, it is important to establish reliable predictive biomarkers along with the treatment strategy, and pathologists are trying to further deduce the implication of spatial characteristics such as intratumoural heterogeneity and immuno-architectures [11,12]. Second, the immune activity is regulated by a complex network of signalling pathways involving many more potential therapeutic targets [6]. Without better understanding of how the network functions as a whole in a quantitative manner, it is difficult to predict the comprehensive outcome of targeting one of these immune checkpoint proteins, or their combinations. In addition, other different types of cancer treatments may be synergistic when combined with immune checkpoint inhibitors, such as radiotherapy, chemotherapy and other immunotherapies including chemokine therapy, adaptive cell transfer and cancer vaccines [13,14]. However, due to the vast number of possible combinations, it is necessary to screen for the ones that are most likely to be effective so that limited resources can be focused on those. Without such predicting power, development of immune checkpoint inhibitors and their combinational therapies is largely a trial-and-error process, and findings in model animals may be difficult to translate well to human subjects [15]. To address the aforementioned problems and further exploit the potential of immune checkpoint inhibitors, it is essential to put together a knowledge base of how the immunotherapies mechanistically modulate the complex interactions between tumour development and anti-tumour immune response. This knowledge base should then allow us to make quantitative predictions of the differential efficacy of an immunotherapy in a heterogeneous population, as well as in combination with other therapeutic agents.

A computational multiscale hybrid model suits this task. Mechanistic dynamical systems models described by ordinary differential equations (ODE) have been developed to understand the dynamics of cancer and immune cells in various situations, including immunotherapy [16–25] (reviewed in [26,27]). However, these models lack the spatial resolution that would allow one to examine intratumoural heterogeneity and their correlation with treatment efficacy. Recently, intratumoural heterogeneity has become centre stage for understanding such important aspects of cancer treatment as drug resistance and biomarkers [28,29]. Agent-based models (ABM) are often employed to capture the spatial aspect of the system. This type of model usually operates on a lattice, where cellular ‘agents’ interact locally with each other according to a defined set of experiment-based rules reflecting their biological roles, and collectively generates a global emergent behaviour. ABM have been used to study many different aspects of cancer, including tumour growth, cancer cell migration, metabolism, evolutionary dynamics, metastasis, angiogenesis and the role of cancer stem cells [30–39]. In particular, Castiglione *et al*. extended a previously developed immune simulator to model cancer–immune interactions, which generated insights into cancer vaccine development [40,41]. Off-lattice ABM are often used when forces between cells or cells and the extracellular matrix play important roles, or change of cell size and morphology need to be accounted for [42–44]. ABM can also serve as the backbone of a hybrid multiscale model by providing a scaffold to interface with other model types to study tumour growth dynamics. Wang *et al*. combined a tumour growth ABM with mechanistic model described by ODE capturing intracellular signalling pathway of epidermal growth factor and transforming growth factor-β, as well as partial differential equation (PDE)-based models describing the diffusion of these chemical substances along with oxygen and glucose [45]. Such scheme is also used in cancer progression models with an immune component, where nutrients for survival and proliferation [46] or an immune reagent [47] are captured as PDE and coupled with the cancer growth ABM.

In this study, we present a computational multiscale agent-based model capturing spatially explicit dynamics of tumour development in the presence of adaptive immune response. By varying components of patient tumour neoantigen characteristics such as mutational burden and antigen strength, our simulations reproduce a variety of spatial patterns of PDL1 expression resembling immuno-architecture found in patient biopsies. We also demonstrate, as a proof of principle, how the model could allow us to predict a scoring system that takes into account not only the relative amount but also the spatial distribution of PDL1^+^ cancer cells and discerns patients that are likely to respond to anti-PDL1 treatment. The model can be expanded to reflect specific types of cancer to enable quantitative predictions of therapy–biomarker combinations and to be used as a platform for conducting virtual clinical trials.

## 2. Material and methods

### 2.1. Hybrid multiscale systems immuno-oncology model

**Figure 1.**
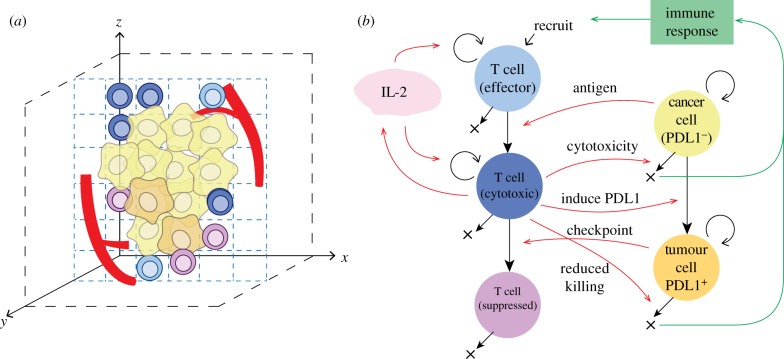
Agent-based model. (*a*) Cellular and structural agents (such as tumour vasculature) are simulated on a three-dimensional grid, with their behaviour defined by a set of rules. (*b*) Rules for cellular agents. T cells are recruited from tumour vasculature, and are all in an effector state upon recruitment. When an effector T cell encounters cancer cells, they are further activated and become actively cytotoxic. Cancer cells start as PDL1^−^ in this model. An activated T cell can kill PDL1^−^ cancer cell with a specified probability once they come close to each other; otherwise, this PDL1^−^ cancer cell can convert to PDL1^+^ with a specified probability. A PDL1^+^ cancer cell can inhibit an activated T cell on contact, and the latter will convert to suppressed state, unable to kill cancer cells. PDL1^+^ cancer cells also exhibit reduced killing probability from activated T cells. Except for suppressed T cells, cells in all the other states can proliferate, and activated T cells can secrete IL-2 which further drives T-cell proliferation. Dead cancer cells will drive immune response, which determines effector T-cell recruitment rate. See Material and methods section for further details.

We developed a multi-compartment multiscale model to capture dynamics of biological processes involved in tumour development and anti-tumour immune response. In this model, we included cytotoxic T lymphocytes (CD8^+^ T cells) and cancer cells, which interact in a three-dimensional space under a set of rules including division, migration, cytotoxic killing and immune evasion. The system includes events at cellular-tissue scale (e.g. TME heterogeneity and immuno-architecture) captured with ABM, as well as molecular scale (e.g. cytokine secretion and transport) captured with PDE. The rules regarding interactions between agents are summarized in figure 1, with further details discussed in the electronic supplementary material.

The ABM is implemented in C++ and simulations run on Linux and Microsoft Windows Operation systems. The diffusion equation for IL-2 is solved with an alternating direction explicit algorithm [48]. Simulation time depends on model parameters including lattice size, cell number and number of time steps to simulate; a typical simulation takes approximately 1 h on a single core of an Intel i7-920 CPU. Model visualization is performed with Amira (FEI, Hillsboro, OR, USA).

We used Johns Hopkins-based Maryland Advanced Research Computing Center (MARCC) for simulations involved in this project, including for parameter sweep and sensitivity analysis.

### 2.2. Simulated immune checkpoint inhibitor treatment

We alter parameter values governing T-cell suppression to represent the effect of anti-PDL1 treatment. Tumour development is simulated as ‘untreated’ initially. After reaching the time point when immunotherapy begins, we reduce the probability of cytotoxic T cell being suppressed by a PDL1^+^ cancer cell by a factor of *m*_supp_ and keep the simulation going. This is to reproduce the scenario when cancer cell surface PDL1 molecules are blocked by an anti-PDL1 mAb and unable to bind to PD1 on effector T cell and induce their exhaustion, yet the blocking is incomplete due to other redundant ligand such as PDL2.

### 2.3. Tumour spatial characterization

At chosen time points during the simulation, we record the status of each voxel, including the type of cell occupying it. If the voxel (*x*, *y*, *z*) is occupied by a cancer cell of any state, we define its label as *L*_raw_(*x*, *y*, *z*) = 1, and otherwise *L*_raw_(*x*, *y*, *z*) = 0; here *x*, *y*, *z* are integers in the range of [0, *N*−1]. We then use an unweighted moving average algorithm to smooth the three-dimensional image. The box edge length is set to three voxels, and the lattice is zero-padded (*L*_raw_(*i*, *j*, *k*) is set to 0 when (*i*, *j*, *k*) exceeds lattice boundaries). The threshold is chosen as 0.5:

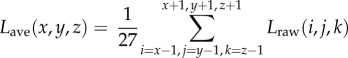


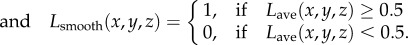
We then apply connected-component labelling to *L*_smooth_ to determine the tumour region. This is done by first finding the non-tumour region, which is defined as the set of voxels with *L*_smooth_ = 0 and which is connected to the edge of the lattice through a path consisting of all non-tumour voxels. Then the complement set is defined as tumour.

When tumour boundary is defined, three cross sections of the tumour are taken with planes perpendicular to the *x-*, *y-* and *z*-axes, respectively, and through the centre of the lattice at (⌊*N*/2⌋, ⌊*N*/2⌋, ⌊*N*/2⌋). For each cross section, we calculate the Euclidian distances of each point within the boundary to the surface of the tumour. Features of cell spatial distribution are then analysed based on their distance to the tumour boundary.

### 2.4. Parametrization and sensitivity analysis

The goal of this study is to develop a computational modelling platform to study the spatial characteristics and spatial heterogeneity of tumour immuno-architecture and its response to immunotherapy. We do not limit our model to represent any specific cancer type at this stage, thus model calibration and parametrization is qualitative at this stage. Values of parameters were taken from experimental studies or adopted from previous models if they apply to a range of cancers. For those parameters for which we are unable to find an experimental value, or those that differ across cancer types, we estimate a range based on best biological knowledge and use global sensitivity analysis to study how biological mechanisms affect simulation outputs.

In sensitivity analysis, we determine the list of parameters of interest and specify a range of values for each parameter (electronic supplementary material, table S1). We use Latin hypercube sampling (LHS) [49] to generate the parameter value combinations to achieve high accuracy with a smaller number of samples. In this study, 500 experiments are generated, with each experiment simulated using one parameter combination from the matrix, and replicated three times to reduce uncertainty from inherent stochastic variations. Partial rank correlation coefficients (PRCC) are calculated between all pairs of target model output and parameters varied in the LHS in order to assess parameter global sensitivity and detect monotonic relationship between mechanisms and functionalities [50].

## 3. Results

### 3.1. Tumour development in the presence of adaptive immune response

**Figure 2.**
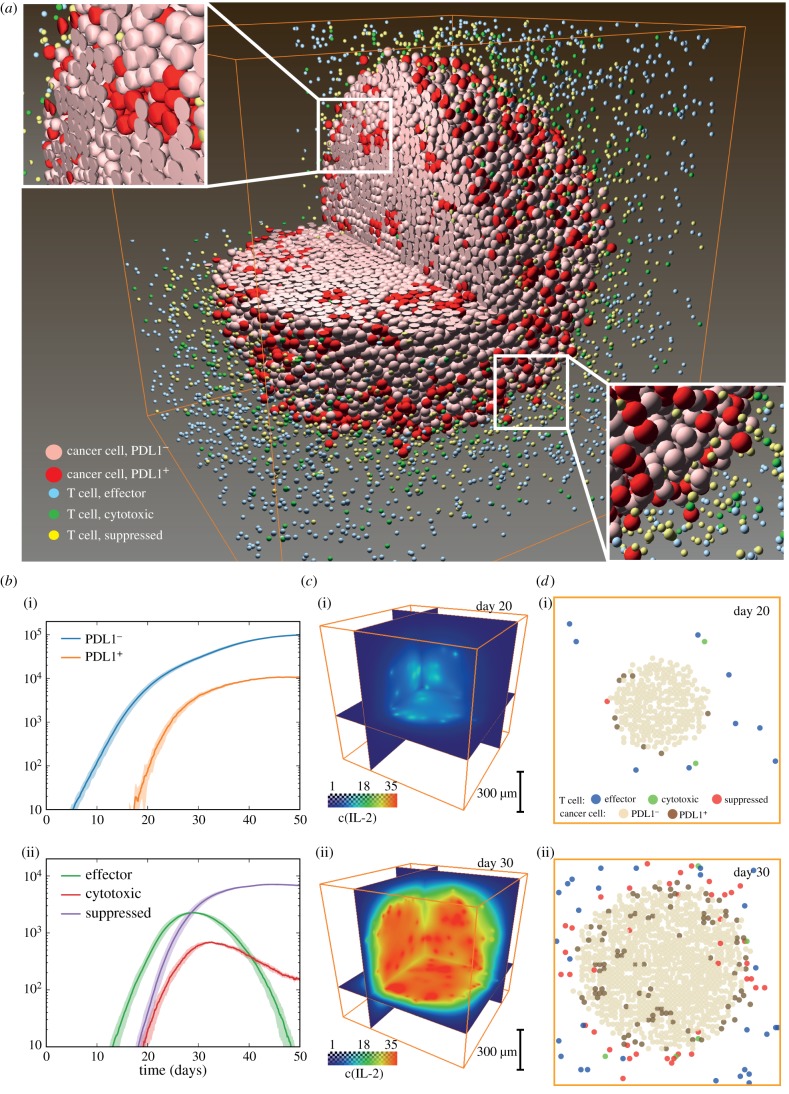
Tumour development baseline. (*a*) Three-dimensional distribution of different cell subsets. Insets show relative locations of T cells and cancer cells in the interior and at the tumour boundary. (*b*) Time courses of cancer and T-cell statistics tracked during simulation. (i) PDL1^+^ and PDL1^−^ cancer cell counts. Shadow indicates ± standard deviation. (ii) Effector, cytotoxic and suppressed T-cell counts. Shadow indicates ± standard deviation. (*c*) Distribution of IL-2 concentration on days (i) 20 and (ii) 30. (*d*) Slices of simulated tumour at days (i) 20 and (ii) 30, showing distribution of different cell types. Orange box is simulated space of 1 mm edge lengths.

We first attempted to establish a baseline scenario where a cancer cell develops into a tumour with relatively stable size while interacting with T cells generated from an immune response induced by cancer progression. Note that here we do not focus on the effects of cancer stem and progenitor cells on tumour growth; these issues are addressed by numerous computational studies, e.g. [37,51]. In addition to the spatial distribution of different cell types at a given time point (figure 2*a*), our model produces output data of various forms for further evaluation and interpretation. Global characteristics such as number of cells of different subtypes (effector, active cytotoxic and suppressed T cells, PDL1^+^, PDL1^−^ cancer cells) are recorded (figure 2*b*). After primed in the lymph node, tumour neoantigen-specific naive T cells differentiate into effector T cells, which can be recruited to tumour. These effector cells begin to exert their cytotoxic activity upon recognizing their antigen target in the TME. As shown in these figures, T cells start to arrive at the tumour site around day 10, quickly accumulate while cancer cell proliferation slows down from exponential growth. This is followed by the appearance of PDL1^+^ cancer cells induced by the inflammatory microenvironment. The number of effector T cells falls after initial growth, leaving behind a mixture of actively cytotoxic and suppressed T cells, and PDL1 positive and negative cancer cells. Molecular scale data are recorded in both space and time. In figure 2*c*, the concentrations of IL-2 at specified time points are visualized, and can be seen to evolve along with tumour progression. To look more closely at how these different subtypes of cells are distributed in space and time, we present slices of the simulated tumour at a series of time points. Each cell subtype is shown with different colours (figure 2*d*), mimicking multiplex immunohistochemistry slides from patient samples [10,52,53]. From these figures, we can discern an immune front formed between immune cells and cancer, with PDL1 positive cancer cells and PD1 positive T cells clustering at the interface; however, patches of these cells also appear inside the tumour. Parameter values for tumour development baseline are listed in electronic supplementary material, table S1. Ten replications are performed for the simulations in this case. It should be noted that these simulations illustrate the power and capabilities of the model platform and the parameters or their ranges should be selected for specific cancer type; this includes the rate of tumour growth that could be much slower than the above example for some cancer types.

### 3.2. Simulated tumours display a range of histological patterns resembling immunohistochemistry data from patient biopsies

**Figure 3.**
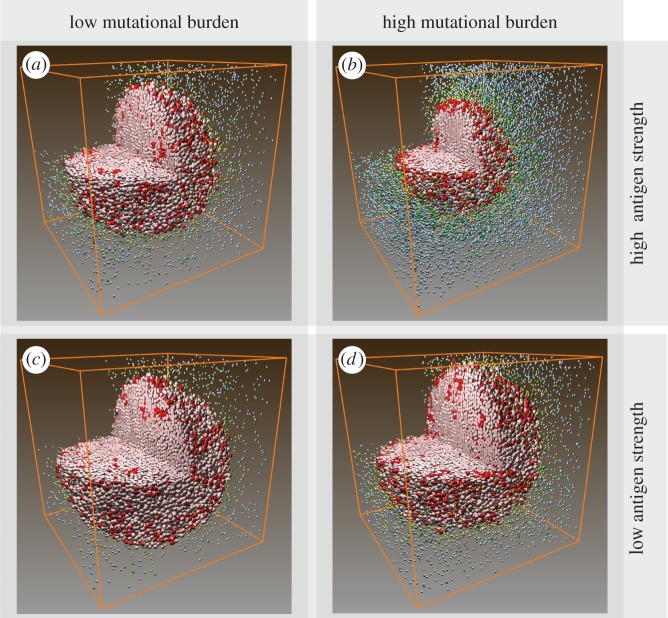
(*a*–*d*) Three-dimensional distribution of cancer cells and T cells in simulated pretreatment tumours among patients with different tumour neoantigen characteristics at day 30.

**Figure 4.**
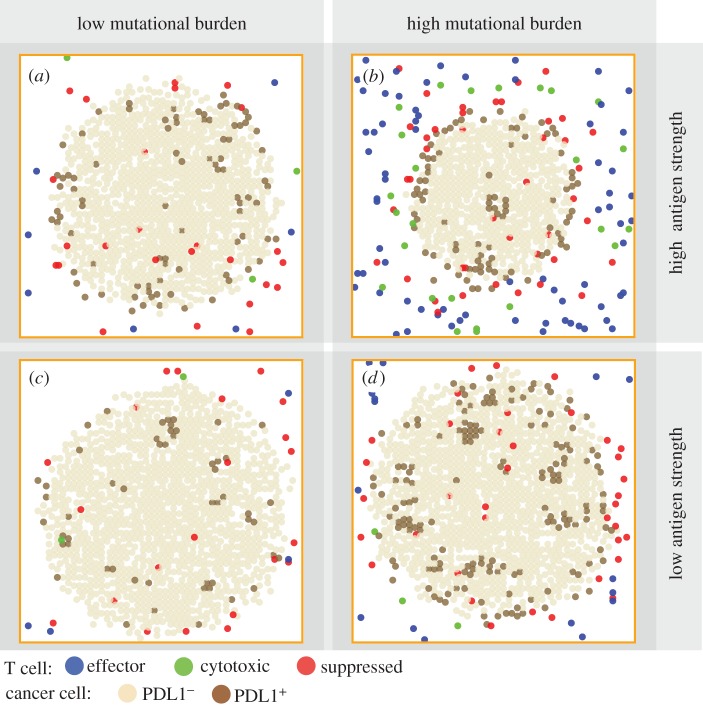
Individual variation of pretreatment patterns among simulated tumours in patients with different tumour neoantigen characteristics at day 30. A tumour slice from a patient with (*a*) tumour neoantigens of low mutational burden and high antigen strength; (*b*) high mutational burden and high antigen strength; (*c*) low mutational burden and low antigen strength; (*d*) high mutational burden and low antigen strength.

Patterns in figure 2*d* represent only one of the many different types of patterns seen in patients' biopsies. Next, we qualitatively validate the model by producing a collection of tumours with a range of patterns with cell type distribution resembling those seen in patients [54–56]. We hypothesize that the patterns can be affected by each individual's tumour neoantigen profile. In our ABM, tumour neoantigen profile is characterized by two factors: mutational burden (*k_a_*) and antigen strength (*k_i_*). These two factors together determine effector recruitment rate in the model (see Material and methods section for details). We simulated tumour development in patients with the following tumour neoantigen characteristics combinations: high (*k_a_* = 20) or low (*k_a_* = 10) mutational burden, with high (*k_i_* = 0.1) or low (*k_i_* = 0.001) antigen strength. Three-dimensional visualizations of tumour at day 30 are shown in figure 3. To better relate our simulation to patient biopsies, available to pathologists, we took snapshots of pretreatment tumour slices at day 30 (figure 4).

We find that different patterns of PDL1^+^ cancer cells emerge from these settings, qualitatively similar to those seen in patients [52,53]. In cases with low mutational burden (figure 4*a*,*c*), PDL1^+^ cancer cells can be seen sporadically distributed within the tumour, and PDL1^+^ cancer cells are seen more frequently in high antigen strength case than in low antigen strength case. When mutational burden is high, PDL1^+^ expression is observed in patchy patterns (figure 4*d*). When antigen strength is also high a clearer immune front is observed, as a PDL1^+^ cuff forms to envelope the tumour. The spatial characteristics (immuno-architecture) have potentials of predictive power of treatment outcomes when quantified [57], which we will further examine in §3.4.

To make the simulations more tractable, the lattice we use to simulate tumour development is 1 mm^3^ in size (i.e. 1 million voxels), which is smaller than actual vascularized tumours; the region could be considered as representative of sub-regions of larger tumours. Electronic supplementary material, figure S1, shows that when larger lattice is used (2 × 2 × 2 mm or 8 million voxels), the overall patterns are similar to those obtained from smaller lattices. While the model does not place limitations on tumour size, the implementation could be limited by the size of the computer RAM, or would be slowed down when the size exceeds a certain computer-specific limit. However, different solutions could be found to circumvent this problem, e.g. considering sample volumes of the tumour.

### 3.3. Responsiveness to anti-PDL1 treatment is affected by patient neoantigen characteristics

**Figure 5.**
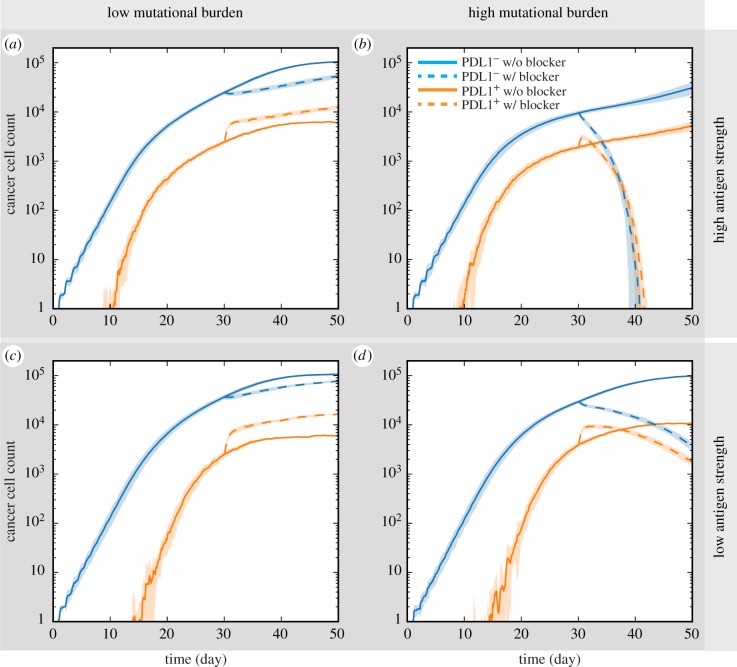
Responses to PD1/PDL1 checkpoint blockade among simulated tumours. Solid line: no treatment. Dotted line: anti-PDL1 treatment is simulated by reducing cytotoxic T-cell suppression from PDL1^+^ cancer cells by half from day 30 onward. Time courses of PDL1 positive and negative cancer cell counts are shown for tumours from patients with (*a*) tumour neoantigens of low mutational burden and high antigen strength; (*b*) high mutational burden and high antigen strength; (*c*) low mutational burden and low antigen strength; (*d*) high mutational burden and low antigen strength.

In a recent study conducted among a colorectal cancer cohort, the mutational burden of patients is found to correlate with response to immune checkpoint blockade treatment [56]. In our next simulation experiments, we attempted to verify that our model reproduces such phenomenon and further predicts which of the two factors, mutational burden versus antigen strength, of a patient's tumour neoantigen profile more strongly influences the responsiveness of the patient to immune checkpoint inhibitor treatment. Here we assume that all tumour cells are equally affected by the treatment, and simulated anti-PDL1 treatment by reducing the parameter value governing the probability of a PDL1^+^ cancer cell suppressing a cytotoxic T cell by *m*_supp_ = 0.8 after day 30 of each simulation. This takes into account the potential redundancy of signalling pathways and incomplete blocking. Results are shown in figure 5. Without treatment, tumours in all four scenarios are progressing from day 30 to day 50. In low mutational burden scenarios, tumour continues to grow with anti-PDL1 treatment regardless of tumour antigen strength. The number of PDL1^−^ cancer cells is smaller than in the cases where treatment is not given, but still increases nevertheless. The number of PDL1^+^ cancer cells is even higher than in no-treatment cases, possibly induced by a more inflammatory TME resulting from reduced suppression of cytotoxic T cells. In high mutational burden scenarios, despite a brief initial increase of PDL1^+^ cells, cancer cell counts begin to decline after treatment is applied after day 30. Furthermore, in high mutational burden cases, the rate of tumour size reduction is affected by antigen strength. With high antigen strength, the tumour is eliminated before the end of simulation (figure 5*c*), while the reduction is less dramatic in the low antigen strength case (figure 5*d*).

### 3.4. Scoring simulated tumour immuno-architecture as a potential biomarker for anti-PDL1 treatment efficacy

Comparing the results in figures 4 and 5, we find that apparent visual similarities may not be indicative of similar prognosis for immune checkpoint blockade treatment. In figure 4*a*,*d*, both tumours appear to have similar sizes and both have PDL1^+^ cancer cells similarly distributed in the tumour. However, the treatment outcome is very different (figure 5*a*,*d*). We use the model to demonstrate how simulated tumour cross sections can be analysed to assess potential predictive biomarkers.

**Figure 6.**
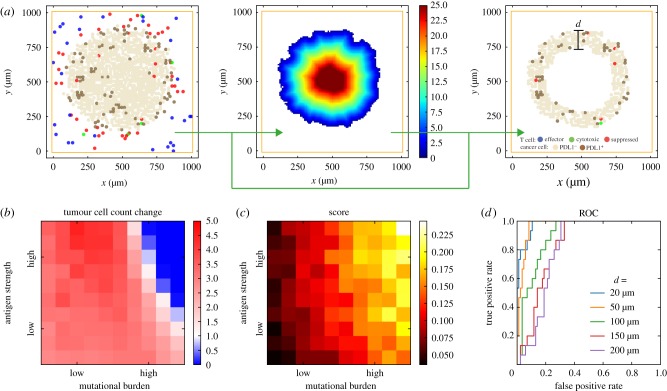
Regionalization and scoring simulated tumour. (*a*) Tumour boundary is detected from spatial distribution of cells from simulation, and a Euclidian distance map is calculated for the distance to surface from every point inside the tumour. Sub-regions can be selected by thresholding the distance of interest (100 µm is used in this example). Regionalized PDL1 score as a potential biomarker for anti-PD1 treatment. (*b*) Simulated treatment outcome. Colour indicates reduction in total cancer cell count at day 50 with anti-PDL1 treatment versus day 30 (pretreatment). (*c*) Score obtained by regionalizing tumour, calculated as the fraction of PDL1^+^ cancer cells within the rim of designated thickness. 50 μm distance cut-off is used in this figure. (*d*) ROC curves of different cutoff distances.

Following the ideas of exploratory scoring systems proposed by pathologists [11,12], we focus on the rim of the tumour, which is defined as the region consisting of points within a specified distance from the tumour surface (figure 6*a*). The distance threshold can be varied in future analyses to optimize the power of the predictive biomarker. For each simulated tumour, three cross sections are taken, perpendicular to the *x*-, *y*- or *z*-axis, and all three go through the centre of the lattice. Euclidian distance to surface is calculated, and for each cross section a score is calculated as the fraction of cancer cells in the rim region that are PDL1 positive. We calculated the ratios of tumour sizes at day 50 (20 days post-treatment) and day 30 (pretreatment) as a measurement of tumour shrinkage (1: equilibrium; 0: elimination; >1: progression) (figure 6*b*). The scoring results using a threshold distance of 50 µm are shown in figure 6*c*.

To assess how the width of evaluated rim affects predictive power of score, we examined a series of threshold distances for regionalization and calculated the receiver operating characteristic (ROC) curve (figure 6*d*). Results indicate that when smaller distance thresholds (e.g. 20 and 50 µm) are used as opposed to larger ones (100+ μm), the score has a higher sensitivity in separating responders from non-responders. The difference is most prominent when high specificity is required for a given sensitivity level, i.e. when we need low false positive rate when using this biomarker to detect responders.

### 3.5. Tumour growth is insensitive to spatial distribution of T-cell entry points

**Figure 7.**
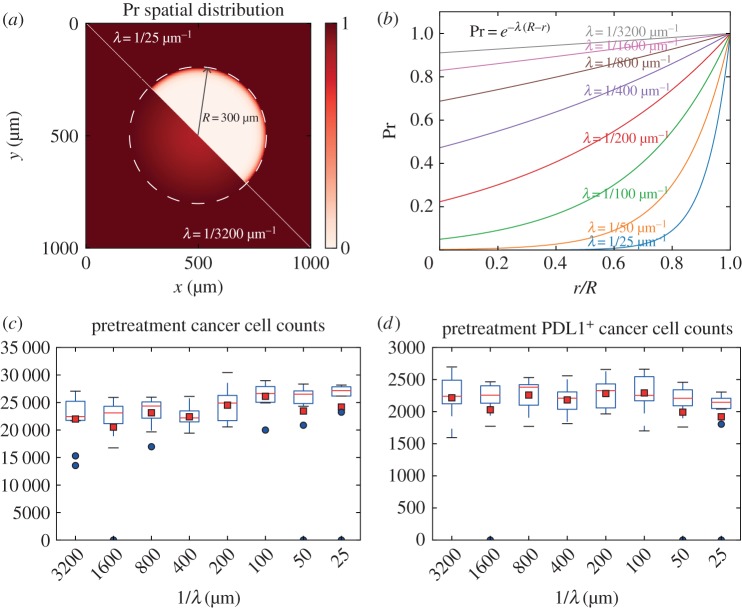
Tumour development shows insensitivity to distribution of T-cell recruitment points. (*a,b*) Parameter *λ* is varied from 1/3200 to 1/25 µm^−1^ to control how fast the density of T-cell entry points drops going inward from the boundary (300 µm). (*c*) Cancer cell counts at day 30 plotted versus 1/*λ*. Red squares indicate mean and blue dots indicate outliers (1.5 IQR or more outside of Q1 or Q3). (*d*) PDL1^+^ cancer cell counts at day 30 plotted versus 1/*λ*.

**Figure 8.**
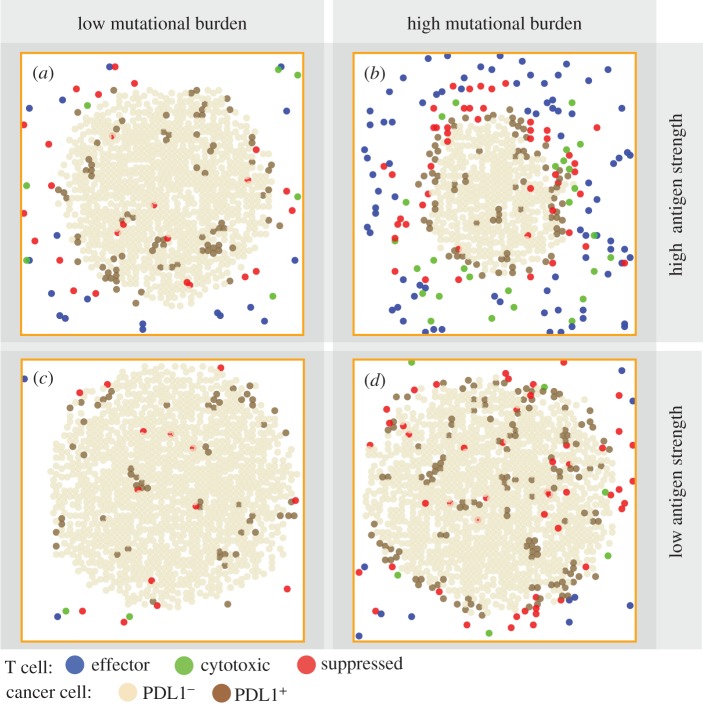
(*a*–*d*) Individual variation of pretreatment patterns in case where simulated tumour core is well perfused (*λ* = 1/1600 µm^−1^). Snapshots represent tumour cross sections at day 30 from patients with different tumour neoantigen characteristics.

In previous sections, we fixed effector T-cell entry point density. However, the arrangement of tumour vasculature may have an impact on tumour growth by affecting T-cell recruitment probability in different regions of a tumour. We varied the parameter governing how steep the drop in vascular density is towards the core (λ) over two orders of magnitude, resulting in cases ranging from almost uniformly distributed (*λ* = 1/3200 µm^−1^) entry points to nearly no entry points in the core (*λ* = 1/25 µm^−1^). Then we compared total cancer cell counts and PDL1^+^ cancer cell counts generated with different vasculature density distributions at a pretreatment (day 30) time point. Ten replications of simulations are performed with each parameter setting. The spatial distribution of recruitment entry (in two dimensions) and resulting cancer cell counts are shown in figure 7. It appears that no obvious correlation exists between *λ* and pretreatment total cancer cell counts or PDL1^+^ cancer cell counts. We also looked into the spatial distribution of PDL1^+^ cancer cells with different neoantigen characteristics when the core of the tumour is well perfused (*λ* = 1/1600 µm^−1^), resulting in nearly uniform distribution of T-cell entry points throughout the tumour. The results are shown in figure 8. We can see that those patterns are similar to those we previously obtained from simulated tumours with relatively poorly perfused cores (figure 4, *λ* = 1/100 µm^−1^).

However, it should be noted that this result may only be relevant to T-cell recruitment locations in tumour. Tumour vasculature is not only responsible for transporting tumour antigen specific T cells; it also delivers oxygen, nutrients, growth factors and therapeutic agents to the tumour. The aforementioned results do not take these factors into account, while the spatial arrangement of tumour vasculature is likely to influence tumour development by shaping the distribution of those factors.

### 3.6. Correlating pretreatment tumour properties with other mechanisms

**Figure 9.**
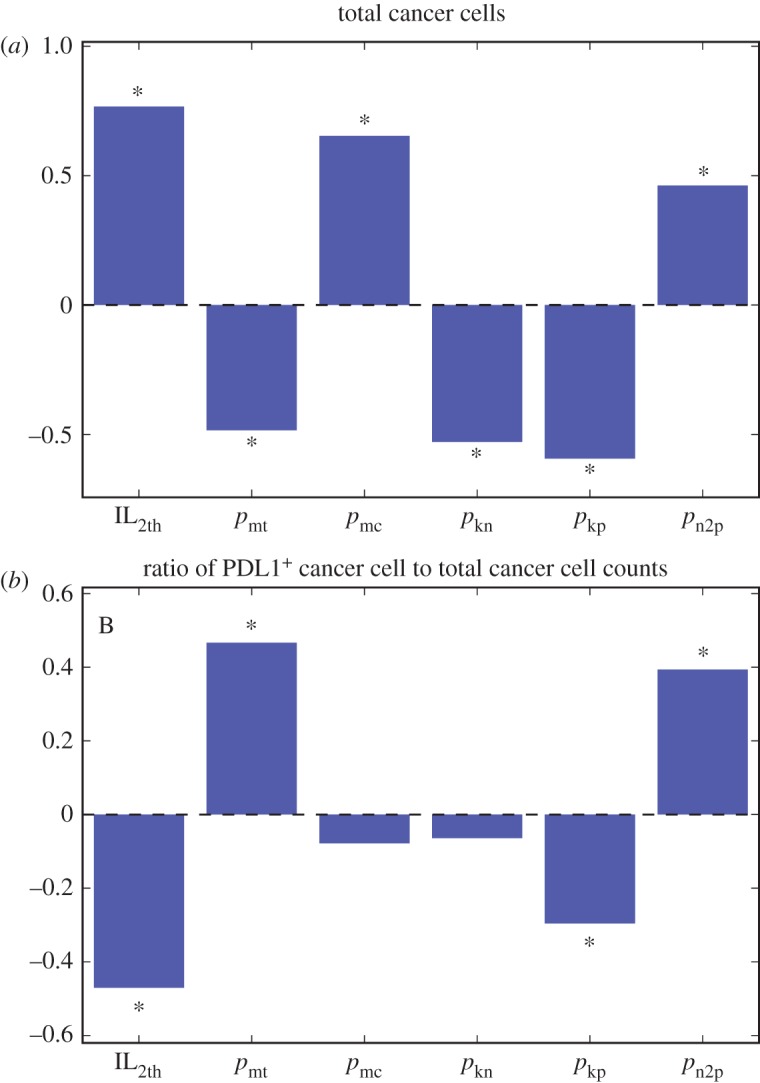
Partial rank correlation coefficients of indicated parameters (for parameter definitions see electronic supplementary material, table S1) and cancer cell count (*a*) or ratio of PDL1^+^ cancer cell to total cancer cell counts (*b*). Significant correlations are marked with asterisks (*α* = 1 × 10^−9^). (Online vesion in colour.)

In §3.3, we analysed the impact of patient neoantigen profile on treatment outlook. For other mechanisms that are parametrized in our model, we use sensitivity analysis to determine the correlation between their values and tumour progression. Parameters included in the analysis are listed in electronic supplementary material, table S1. The parameters with significant correlation with pretreatment tumour size/total cancer cell count and the ratio of PDL1^+^ cancer cell to total cancer cell counts are shown in figure 9, along with their PRCC values.

With regard to total cancer cell count, the results show that the IL-2 threshold to activate effector T-cell division is positively correlated with pretreatment tumour size, indicating the important role of anti-tumour effector T-cell proliferation in TME to limit tumour size without immunotherapy. T-cell motility is negatively correlated with total cancer cell count, meaning an increase in ability of highly mobile CD8^+^ T cells to limit tumour growth. The velocities of cancer cells vary in a wide range (0.01–5 µm min^−1^) depending on cancer types, stages of progression and migration modes [58]. In this study, we are focusing on the lower end of the spectrum corresponding to properties found in the early stage of tumour development. At this stage, cancer cell mobility is positively correlated with tumour size, indicating cancer cell migration promotes tumour progression. The negative correlation between tumour size and rate of cytotoxic T cells killing both PDL1^+^ and PDL1^−^ cancer cells is also in accordance with the role of anti-tumour T cells in limiting cancer growth. The higher correlation of the killing rate of PDL1^+^ than that of PDL1^−^ also suggests the importance of a higher PDL1^+^ cancer cells' overall contribution to tumour progression. This will help us assess their correlation with treatment efficacy and thus potentials as target of clinical intervention. PDL1 induction in cancer cells also correlates positively with tumour size. This indicates that the resistance build-up from the inflammatory environment not only prevents a tumour from been eliminated by the immune system but also contributes to its progression.

Furthermore, some mechanisms are correlated with both total cancer cell count and PDL1^+^ fractions, but in opposite directions. A high proportion of PDL1^+^ expression in tumours is usually considered to suggest high probability of patient responding to anti-PD1/anti-PDL1 treatment, and thus mechanisms that correlate negatively with tumour size and positively correlate with PDL1^+^ expression might be helpful to couple with such treatment to improve outcome. From figure 9*b*, it can be seen that IL-2 threshold belongs in such category, meaning sensitizing T cells to IL-2 may synergize with the therapy. Alternatively, mechanisms negatively correlated with tumour size and positively correlated with PDL1^+^ fraction, such as T-cell motility, can be induced to help with the therapy. On the contrary, targeting mechanisms such as cancer cell PDL1 induction probability might not be fruitful because although increasing it can promote PDL1 fraction, it also promotes overall cancer growth, leaving the overall effect difficult to predict.

## 4. Discussion

In this study, we developed a three-dimensional agent-based systems immuno-oncology model to simulate the interaction between cancer cells and CD8^+^ T cells specific to cancer antigen. The inputs of each simulation to capture individual variance are parameters governing the tumour mutational burden and antigen strength. Two important model outputs are assessed. First, the emergent spatial patterns of different cell types from our simulations without treatment, which resemble patterns observed in patients' biopsies. Second, the treatment outcome of simulated immune checkpoint blockade therapy for each patient. By comparing the input and the emergent patterns in pretreatment tumour, we attribute the source of tumour spatial heterogeneity to patient tumour neoantigen profile. By comparing the two outputs, namely pretreatment immuno-architecture and treatment outcome, we presented an *in silico* framework to assess potential predictive biomarkers for the treatment by correlating simulated treatment outcome with metrics obtained by applying the scoring scheme on cross sections from simulated tumour.

At its current stage, the model can qualitatively capture characteristics of a spectrum of cancers and is able to assess and compare predictive biomarkers in a semi-quantitative manner. However, at this stage it is not calibrated to any particular type of cancer, which prevents it from generating predictions that can directly be used in clinical practice. By fitting the model parameters to a specific type of cancer, the model will transform into a platform for hypotheses testing. The data required for model calibration would include tumour biopsies from cancer patients, and preferably with corresponding post-treatment outcome. Such dataset can be used to both quantify different cell types as well as record their spatial arrangement. Half the images can be considered training data and be used to calibrate the ABM so that simulated treatment outcome will match with patient treatment outcome, while the other half can serve as testing data reserved to check if the model predicts treatment outcome with reasonable accuracy that can be statistically assessed. Once this is done, the same dataset can be used again in biomarker discovery, as a standard to validate the power of biomarkers that are predicted by the computational model.

Several limitations should be noted for our current model. First, the lattice set-up constrains the flexibility of cell diameters. Making all cells on-lattice means that diameters of cells can only be multiples of the smallest cells being simulated, which in this case are CD8^+^ T cells. In the current model implementation, we set the diameter of T cells to be 10 µm and the diameter of cancer cells to be 20 µm to reflect their relative size. While this is a reasonable assumption, cancer cells can be of different sizes depending on the type of cancer and the size can follow a distribution. To relax this assumption, we can use an off-lattice setting and make the diameter of cells a continuous variable. This will increase the computational power required when we simulate the dynamics, because collision detection in an off-lattice space requires extra calculations and memory space. One potential benefit of this choice is that we can then divide the simulation space into sub-regions and parallelize the computational process. However, because we always need to perform global sensitivity analysis to address parameter uncertainty, the large number of simulations required can cancel out the benefit of parallelization of each individual simulation. Nonetheless, if the constraint on cell size does affect the accuracy of the model's predictive power, an off-lattice scheme has to be used despite the extra computational time.

The current version of the model is also limited in that some of the modules are coarse-grained or phenomenological. At molecular scale, interactions of PD1/PDL1 on T cells and cancer cells are rule based and lack explicit mathematical formulation of receptor–ligand dynamics. The effect of anti-PDL1 antibody works through changing the immunosuppression probability. ODE modules can be employed to capture receptor–ligand dynamics and signal transduction in cancer cells and T cells, as well as pharmacokinetics (PK) and pharmacodynamics (PD), in order to quantitatively model effects of therapeutic agents and how tumours respond to treatment. At the cellular-tissue scale, some important cell types found in the TME are not included in the current model, such as regulatory T cells and myeloid-derived suppressor cells. Regulatory T cells can inhibit tumour immune response at multiple stages, including antigen presentation, T-cell priming and expansion in lymph nodes and T-cell activity in the tumour lesion. They potentially contribute to the resistance of tumour against the immune response. We are evaluating the implication of such interactions in a molecularly detailed systems pharmacology model [59], and such mechanisms can also be explored in our spatially resolved paradigm by adding new classes of cells and including pertaining rules. Other important biological factors not currently taken into account include cytokines other than IL-2. The complex crosstalk of cytokines in the TME can be further studied for their implication in tumour heterogeneity and differential responses to treatment by extending the model to include them explicitly. Tumour vasculature in this model is also reduced to entry points of effector T cells, while other important functions of the vasculature are not taken into account. A dynamic tumour vasculature module coupled with tumour perfusion should be able to capture tumour development in a more mechanistic manner. The vascular geometry could either be taken from experiments [60] or from computational models [38,61]. At an organ-system scale, events including CD8^+^ T-cell priming (in the lymph node), trafficking (via blood circulation) and recruitment to the tumour compartment are largely simplified and phenomenological. A more sophisticated lymph node compartment sub-model can substitute current module so that priming is more accurately and mechanistically represented, allowing more stringent interrogation into roles of anti-tumour immune components such as T-cell clonality. The model platform we developed in this study is built in a modular and extendable manner. The aforementioned modules can be replaced with ones with more fine-grained versions as discussed. If such models have been developed by other laboratories, they can be incorporated into the present model via readily available interfaces; otherwise these modules have to be built *de novo* and connected to the core model. Particularly, with the implementation of PK/PD modules and rigorous model calibration and validation, the model can be used as a platform for *in silico* drug discovery and conducting virtual clinical trials [62].

## Supplementary Material

Supplementary Information

## Data accessibility

A GUI version of binary executable (tested on Windows 7 and Windows 10) with parameter file template can be accessed at: popellab.johnshopkins.edu/software/CancerImmuneABM/.
