# Supplementary material for: A computational multiscale agent-based model for simulating spatio-temporal tumour immune response to PD1 and PDL1 inhibition
Source: J R Soc Interface. 2017 Sep 20;14(134):20170320. doi: 10.1098/rsif.2017.0320 (PMC5636269; doi:10.1098/rsif.2017.0320)
Supplement: Supplementary Information [file rsif20170320supp1.docx]

**Supplementary Information for:**

**A computational multiscale agent-based model for simulating spatio-temporal tumor immune response to PD1 and PDL1 inhibition**

**(Journal of the Royal Society Interface)**

Chang Gong^1*^, Oleg Milberg^1^, Bing Wang^2^, Paolo Vicini^3^, Rajesh Narwal^4^, Lorin Roskos^4^, Aleksander S. Popel^1^

^1^Departments of Biomedical Engineering and Oncology, Sidney Kimmel Comprehensive Cancer Center, Johns Hopkins University School of Medicine, Baltimore, Maryland; ^2^MedImmune, Mountain View, California; ^3^MedImmune, Cambridge, United Kingdom; ^4^MedImmune, Gaithersburg, Maryland

*Supplementary Information*

*Agent-based Model rules*

In the present study, we focus on implementing the ABM model of the tumor and using this model to reproduce and investigate the spatio-temporal characteristics of cancer cells as well as tumor microenvironment. The model will include the following cellular and molecular elements: two subtypes of cancer cells: PDL1- and PDL1+; three subtypes of CD8+ T cells: effector T cells, activated T cells, and suppressed T cells; and cytokine IL-2. The elements of the model are illustrated in Fig. 1.

Model agents and interaction rules

**Cancer cells**

In what follows, the rules of the agent-based model are formulated based on the abundant experimental evidence; in this study we aim at building as simple a model as possible, to demonstrate proof of principle. Subsequently, every rule of the model could be modified and extended to accommodate experimental data for a specific cancer type. In the simulations we chose our voxel linear dimension as *l_v_*. Cancer cells have the diameter *d_c_*_._ Two subtypes of cancer cells are considered in this model: PDL1- and PDL1+ cancer cells. At the beginning of each simulation (t=0), one PDL1- cancer cell is initialized at the center of the lattice. At each time step, each cancer cell will move at probability p_mc_, and the destination can be any of the six non-self von Neumann neighborhood directions (the von Neumann neighborhood on a three-dimensional square lattice is composed of a central voxel and six voxels adjacent to the sides of the central voxel). When migrating, cancer cells can push T cells away if their target destinations are blocked by those T cells, in order to account for their size differences. When cancer cells push T cells away, they first identify the T cells to be pushed away, and check their shared Moore neighborhood to verify if there are enough open grid locations for them to move into. If there are enough open locations, T cells are relocated to these openings; otherwise cancer cell will not move to this direction. There is no recruitment of cancer cells in our current model. Cancer cell population expands through proliferation, and both cancer cell subtypes (PDL1- and PDL1+) are allowed to divide for unlimited number of times. Every time after a cancer cell divides, its internal division countdown is reset based on doubling time parameter, and when that countdown reaches 0, it will attempt to create a daughter cell in its Moore neighborhood (the Moore neighborhood is defined here as composed of the central voxel and 26 voxels adjacent to the central voxel, i.e. the voxels having at least one common point) if there are open spaces; otherwise this will happen when the first opening appears. The daughter cell will inherit most of the properties of the original cell, including the PDL1 expression state. One exception is that both cells’ lifespan will be reset by drawing a random number from normal distribution N(µ_c_,σ_c_), where µ_c_ and σ_c_ are mean and standard deviation of cancer cell lifespan, respectively (if a negative number is chosen, it is replaced by 0 and cell dies in the same time step). If a cancer cell exhausts its lifespan (which is drawn when the cell was created), it will also die at the end of this time step.

At each time step, cancer cells interact with their local environment. For PDL1- cancer cells, if any activated T cell is in its Moore neighborhood, this cancer cell will be killed with a probability p_kn_ (PDL1- being killed)_._ If it is not killed, it will convert to PDL1+ state (1–3) with a probability p_n2p_ (PDL1- to PDL1+). For PDL1+ cancer cells, killing occurs with probability p_kp_ when activated T cells present in its Moore neighborhood. At the probability of p_p2n_, PDL1+ cancer cells may also lose PDL1 expression and return to PDL1- state.

**T cells**

T cell has a diameter of *d_t_*; T cells are recruited from the vasculature. Details about T cell recruitment will be discussed later in this section. At each time step, each T cell will move with a probability p_mt_, and the destination can be any of the six von Neumann neighborhood directions if they are unoccupied. When first recruited, T cells are already effector cells as they have gone through the priming process in secondary lymphoid organs. However, they need to contact tumor antigen again through T cell receptor to become fully activated. At each time step, effector T cell scans its Moore neighborhood for cancer cells, and converts to an activated state if it finds any. Activated T cells release cytokine IL-2 into its residing voxel space for a given period of time (*t_il2_*) after they first convert from effector state. The IL-2 concentration is governed by diffusion equation:

$$\frac{\partial C_{il2}}{\partial t}=D_{il2}\nabla^{2}C_{il2}-d_{il2}C_{il2}+\lambda_{il2}$$

Here $D_{il2}$ is the IL-2 diffusion coefficient, $d_{il2}$ is the degradation rate, and $\lambda_{il2}$ is IL-2 production rate which depend on T cell states at each location. Activated T cell also scans its Moore neighborhood for PDL1+ cancer cells specifically, and if found, will convert to a suppressed state at a probability p_supp_. Suppressed T cells remain exhausted before they die (4). Effector and activated T cells can proliferate for a limited number of times, when IL-2 concentration at their location is above a threshold (*IL2_th_*). This criterion is always satisfied by newly converted activated T cells, which will receive sufficient IL-2 via autocrine signaling, and may be satisfied by effector cells in the vicinity of activated T cells via paracrine signaling. Division occurs at fixed time intervals, and daughter cells are generated when opening appears in their Moore neighborhood. The daughter cell will inherit traits including remaining division counts and current cell state, but will reset its lifespan. When T cells reach the end of their lifespan, they will die and be cleared from the space.

T cell recruitment to tumor compartment

After being primed in secondary lymphoid tissues, tumor specific effector T cells traffic with blood circulation before they are recruited to the tumor microenvironment. Thus we assume that the location of their entry to the TME correlates with tumor vessel density and perfusion. In the tumor compartment of the ABM, we generate a set of points as entries for effector T cells according to the spatial characteristics of tumor vasculature. These points serve as sources of T cell recruitment to the tumor microenvironment.

Tumor vasculature is characterized by abnormal and heterogeneous vascular density distribution compared with those of normal tissues (5). As a result, some regions of tumor have a larger distance to nearest vessels compared to normal tissues (6), especially in the core of a tumor as opposed to the rim (7). It is also revealed by imaging (8) as well as hemodynamic models based on tumor xenograft vasculature (9) that perfusion is higher in the rim/periphery region of the tumor, even though well perfused pockets can be found in the core. In order to represent such features and not rely on the detailed angioarchitecture which for most tumor types remains unknown, we randomly generate the set of entry points throughout the lattice and adjust their density based on their location. To simulate T cell recruitment to the tumor with a relatively sparsely vascularized and less perfused core versus a better perfused rim, a total number of N_vas_ entry points are first randomly drawn with a uniform distribution within the boundary of the lattice, and then each potential entry point is generated with a probability determined by the distance to the center of the lattice, *r*:

$$\Pr=\left\{ \begin{aligned} e^{-\lambda\left( R-r \right)}, r<R \\ 1, r\geq R \end{aligned} \right.$$

Here *R* (μm) is the radius of the lower vascular density core while 𝝺 (μm^-1^) determines how sharply vascular density decreases going inwards, towards the center of the tumor. The effect of 𝝺 on model outcome is explored in later sections. Importantly, the model also allows incorporation of the detailed angioarchitecture from imaging data that has recently become available for some tumor xenograft models (9,10), but not yet available for human subjects. Thus, a simplified approach that we use in this study is a necessity.

Module of effector T cell generation in the lymph node and recruitment to the tumor

Effector T cells are recruited from voxels that are labeled as tumor vasculature. Conceptually, we assume the number of T cells entering the simulation at every time step is proportional to blood effector T cell concentration, which in turn is proportional to the number of effector T cells produced by the tumor-draining lymph node. We use a Michaelis-Menten term as a proxy to recruitment rate of effector T cell at time point t, $r(t)$:

$$r(t)= \frac{k_{a}N_{c, death}(t-t_{delay})r_{1}}{\frac{1}{k_{i}}+N_{c, death}(t-t_{delay})}$$

where

$$N_{c, death}\left( t \right)= \sum_{s=t-0.5*t_{window}}^{t+0.5*t_{window}} n_{c, death}(s)$$

Here $n_{c, death}(s)$ is the number of cancer cell deaths at time step *s*, and $N_{c, death}(t)$ is the cumulative death during the $t_{window}$ time period centered at time point *t*. $t_{delay}$ accounts for the time needed in an adaptive immune response for dendritic cell trafficking and T cell priming in the lymph node. $k_{a}$ and $k_{i}$ represent the mutational burden and strength of the tumor neoantigens of a patient, respectively (11). We assume that the mutational burden determines the number of available naïve T cells clones specific to tumor neoantigens (antigen-spreading), which in turn controls the maximum effector T cell blood concentration. The mutational burden varies in a very wide range in different cancer types and among patients with the same type of cancer (12), and in our model it can range from 0 to infinity. Antigen strength represents the average immunogenicity of tumor specific antigens, whose inverse determines how much cancer cell debris (represented by cancer cell death events) is required to elicit a successful immune response; this parameter ranges from 0 to 1. $r_{1}$ is the maximum recruitment rate when tumor mutational burden is 1. Thus, effector T cell recruitment rate is time dependent, affected by cancer cell death as well as the tumor neoantigen profile of a patient. The full list of model parameters and relevant references is presented in Table S1.

Model geometry and discretization

The size of simulation domain can be defined by parameter settings; in general, we simulate tumor development and immune cell activity in tumor contained within a cube of side L. The cube is divided into N cubic voxels of side lv. Time is also discretized into equal time steps. For the agent-based model, time step tstep can be adjusted depending on the temporal resolution required for the biological process of interest.


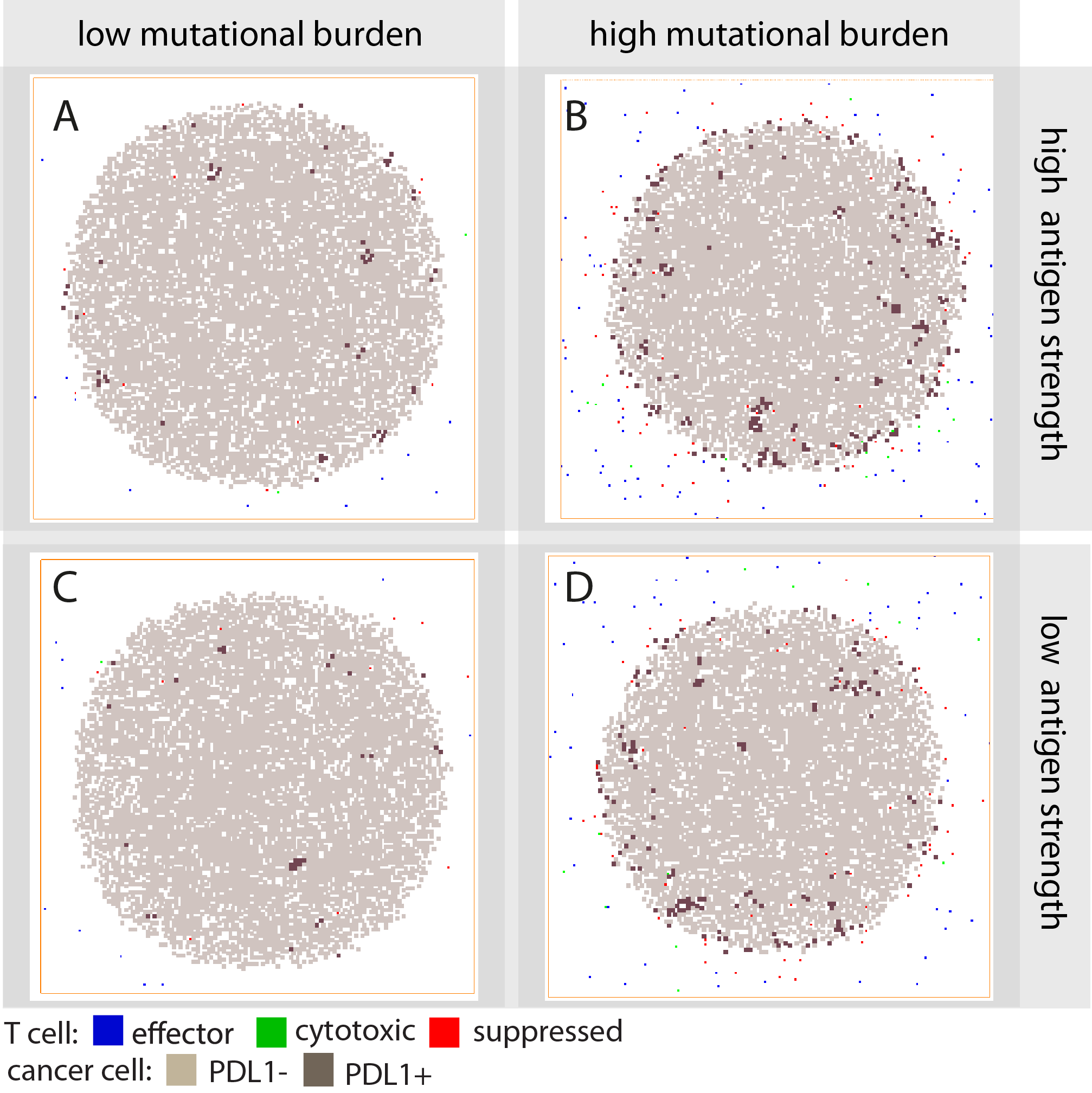


Figure S1. Individual variation of pretreatment patterns among simulated tumors in patients with different tumor neoantigen characteristics at day 50. Tumors are simulated in a 2x2x2 mm lattice (8 million voxels), and the sparse T cell entry core R = 600 μm.

Table S1. Parameter values for baseline tumor development and *in silico* experiments

| category | parameter | baseline | neoantigen  Properties | vessel  distribution | LHS | unit | reference |  |
| --- | --- | --- | --- | --- | --- | --- | --- | --- |
| Environment |  |  |  |  |  |  |  |  |
|  | time step, t_step_ | 10 |  |  |  | min |  |  |
|  | lattice interval, l_v_ | 10 |  |  |  | μm |  |  |
|  | lattice size, N | 101 |  |  |  |  |  |  |
|  | Total entry points, N_vas_ | 5000 |  |  |  | μm | (13)* |  |
|  | Sparse T cell entry core radius, R | 300 |  | [250, 350] |  | μm |  |  |
|  | Sparse T cell entry core transition, 𝝺 | 0.01 |  | [0.002, 0.05] |  | μm^-1^ |  |  |
| T Cell |  |  |  |  |  |  |  |  |
|  | Diameter, d_t_ | 10 |  |  |  | μm | (14) |  |
|  | lifespan Mean, µ_t_ | 3 |  |  |  | day | (15)* |  |
|  | lifespan SD, σ_t_ | 0.5 |  |  |  | day |  |  |
|  | IL-2 threshold for division, IL2_th_ | 800 |  |  | [200, 1000] | pM·hr | (16)* |  |
|  | IL-2 release rate | 17600 |  |  |  | hr^-1^ | (17) |  |
|  | IL-2 release duration, t_il2_ | 12 |  |  |  | hr | (17) |  |
|  | moving probability, p_mt_ | 0.95 |  |  | [0.2, 0.99] | per time step | (18)* |  |
|  | suppression probability, p_supp_ | 0.1 |  |  |  | per time step |  |  |
|  | Antibody blocking of PDL1, m_supp_ | 0.8 | 0.5 |  |  |  |  |  |
|  | division Interval | 8 |  |  |  | hr | (15,19) |  |
|  | max division count | 8 |  |  |  |  | (20)* |  |
|  | recruitment rate base, $r_{1}$ | 1 |  |  |  | per time step |  |  |
| Cancer Cell |  |  |  |  |  |  |  |  |
|  | Diameter, d_c_ | 20 |  |  |  | μm | (21–23) |  |
|  | lifespan Mean, µ_c_ | 5 |  |  |  | day |  |  |
|  | lifespan SD, σ_c_ | 2 |  |  |  | day |  |  |
|  | moving probability, p_mc_ | 0.01 |  |  | [0.001, 0.1] | per time step | (24) |  |
|  | PDL1- death probability, p_kn_ | 0.1 |  |  | [0.05, 0.3] | per time step | (25)* |  |
|  | PDL1+ death probability, p_kp_ | 0.05 |  |  | [0.05, 0.3] | per time step |  |  |
|  | probability to switch from PDL1- to PDL1+, p_n2p_ | 0.8 |  |  | [0.1, 0.9] | per time step |  |  |
|  | probability to switch from PDL1+ to PDL1-, p_p2n_ | 0.02 |  |  |  | per time step | (26)* |  |
|  | division interval | 24 |  |  |  | hr | (27) |  |
|  | neoantigen strength, $k_{i}$ | 0.01 | [0.001, 0.1] |  |  |  |  |  |
|  | mutational burden, $k_{a}$ | 15 | [5, 25] |  |  |  |  |  |
| Immune Response | |  |  |  |  |  |  |  |
|  | Immune response delay  $t_{delay}$ | 5 |  |  |  | day | (28,29) |  |
|  | $t_{window}$ | 1 |  |  |  | day |  |  |
| Molecular |  |  |  |  |  |  |  |  |
|  | IL-2 diffusivity, D_il2_ | 1.00E-07 |  |  |  | cm^2^/s | (17) |  |
|  | IL-2 degradation rate, d_il2_ | 0.1 |  |  |  | hr^-1^ | (17) |  |

Baseline: baseline tumor progression, see section 3.1. Neoantigen properties: varying mutational burden and antigen strength, see section 3.2-3.4. Vessel distribution: distribution of effector T cell entry points, see section 3.5. LHS: Latin hypercube sampling, see section 3.6. Unless otherwise listed, parameter values are the same as used in the baseline scenario. * parameters are estimated from experimental data in the reference.

**References**

1. Lipson EJ, Vincent JG, Loyo M, Kagohara LT, Luber BS, Wang H, et al. PD-L1 Expression in the Merkel Cell Carcinoma Microenvironment: Association with Inflammation, Merkel Cell Polyomavirus, and Overall Survival. Cancer Immunol. 2013 Jul 1;1(1):54–63.

2. Spranger S, Spaapen RM, Zha Y, Williams J, Meng Y, Ha TT, et al. Up-Regulation of PD-L1, IDO, and Tregs in the Melanoma Tumor Microenvironment Is Driven by CD8+ T Cells. Sci Transl Med. 2013 Aug 28;5(200):200ra116-200ra116.

3. Taube JM, Anders RA, Young GD, Xu H, Sharma R, McMiller TL, et al. Colocalization of Inflammatory Response with B7-H1 Expression in Human Melanocytic Lesions Supports an Adaptive Resistance Mechanism of Immune Escape. Sci Transl Med. 2012 Mar 28;4(127):127ra37-127ra37.

4. Butte MJ, Keir ME, Phamduy TB, Freeman GJ, Sharpe AH. PD-L1 interacts specifically with B7-1 to inhibit T cell proliferation. Immunity. 2007 Jul;27(1):111–22.

5. Jain RK. Normalization of Tumor Vasculature: An Emerging Concept in Antiangiogenic Therapy. Science. 2005 Jan 7;307(5706):58–62.

6. Baish JW, Stylianopoulos T, Lanning RM, Kamoun WS, Fukumura D, Munn LL, et al. Scaling rules for diffusive drug delivery in tumor and normal tissues. Proc Natl Acad Sci. 2011 Feb 1;108(5):1799–803.

7. Vakoc BJ, Lanning RM, Tyrrell JA, Padera TP, Bartlett LA, Stylianopoulos T, et al. Three-dimensional microscopy of the tumor microenvironment in vivo using optical frequency domain imaging. Nat Med. 2009 Oct;15(10):1219–23.

8. Bowden DJ, Barrett T. Angiogenesis Imaging in Neoplasia. J Clin Imaging Sci. 2011 Jan 1;1(1):38.

9. Stamatelos SK, Kim E, Pathak AP, Popel AS. A bioimage informatics based reconstruction of breast tumor microvasculature with computational blood flow predictions. Microvasc Res. 2014 Jan;91:8–21.

10. Boujelben A, Watson M, McDougall S, Yen Y-F, Gerstner ER, Catana C, et al. Multimodality imaging and mathematical modelling of drug delivery to glioblastomas. Interface Focus. 2016 Oct 6;6(5):20160039.

11. Brown SD, Warren RL, Gibb EA, Martin SD, Spinelli JJ, Nelson BH, et al. Neo-antigens predicted by tumor genome meta-analysis correlate with increased patient survival. Genome Res. 2014 May 1;24(5):743–50.

12. Alexandrov LB, Nik-Zainal S, Wedge DC, Aparicio SAJR, Behjati S, Biankin AV, et al. Signatures of mutational processes in human cancer. Nature. 2013 Aug 22;500(7463):415–21.

13. Thompson HJ, McGinley JN, Knott KK, Spoelstra NS, Wolfe P. Vascular density profile of rat mammary carcinomas induced by 1-methyl-1-nitrosourea: implications for the investigation of angiogenesis. Carcinogenesis. 2002 May 1;23(5):847–54.

14. Kuse R, Schuster S, Schübbe H, Dix S, Hausmann K. Blood lymphocyte volumes and diameters in patients with chronic lymphocytic leukemia and normal controls. Blut. 1985 Apr 1;50(4):243–8.

15. Boer RJD, Homann D, Perelson AS. Different Dynamics of CD4+ and CD8+ T Cell Responses During and After Acute Lymphocytic Choriomeningitis Virus Infection. J Immunol. 2003 Oct 15;171(8):3928–35.

16. Smith KA. Cell Growth Signal Transduction Is Quantal. Ann N Y Acad Sci. 1995 Sep 1;766(1):263–71.

17. Busse D, de la Rosa M, Hobiger K, Thurley K, Flossdorf M, Scheffold A, et al. Competing feedback loops shape IL-2 signaling between helper and regulatory T lymphocytes in cellular microenvironments. Proc Natl Acad Sci U S A. 2010 Feb 16;107(7):3058–63.

18. Boissonnas A, Fetler L, Zeelenberg IS, Hugues S, Amigorena S. In vivo imaging of cytotoxic T cell infiltration and elimination of a solid tumor. J Exp Med. 2007 Feb 19;204(2):345–56.

19. Altman BJ, Dang CV. Normal and cancer cell metabolism: lymphocytes and lymphoma. FEBS J. 2012 Aug 1;279(15):2598–609.

20. Wong P, Pamer EG. Cutting Edge: Antigen-Independent CD8 T Cell Proliferation. J Immunol. 2001 May 15;166(10):5864–8.

21. Chaffer CL, Weinberg RA. A Perspective on Cancer Cell Metastasis. Science. 2011 Mar 25;331(6024):1559–64.

22. Chambers AF, Groom AC, MacDonald IC. Metastasis: Dissemination and growth of cancer cells in metastatic sites. Nat Rev Cancer. 2002 Aug;2(8):563–72.

23. Wolf K, Wu YI, Liu Y, Geiger J, Tam E, Overall C, et al. Multi-step pericellular proteolysis controls the transition from individual to collective cancer cell invasion. Nat Cell Biol. 2007 Aug;9(8):893–904.

24. Clark AG, Vignjevic DM. Modes of cancer cell invasion and the role of the microenvironment. Curr Opin Cell Biol. 2015 Oct;36:13–22.

25. Pardo J, Bosque A, Brehm R, Wallich R, Naval J, Müllbacher A, et al. Apoptotic pathways are selectively activated by granzyme A and/or granzyme B in CTL-mediated target cell lysis. J Cell Biol. 2004 Nov 8;167(3):457–68.

26. Lee S-J, Jang B-C, Lee S-W, Yang Y-I, Suh S-I, Park Y-M, et al. Interferon regulatory factor-1 is prerequisite to the constitutive expression and IFN-γ-induced upregulation of B7-H1 (CD274). FEBS Lett. 2006 Feb 6;580(3):755–62.

27. Eden E, Geva-Zatorsky N, Issaeva I, Cohen A, Dekel E, Danon T, et al. Proteome Half-Life Dynamics in Living Human Cells. Science. 2011 Feb 11;331(6018):764–8.

28. Central Memory and Effector Memory T Cell Subsets: Function, Generation, and Maintenance. Annu Rev Immunol. 2004;22(1):745–63.

29. Murphy K, Weaver C. Janeway’s Immunobiology, 9th edition. Garland Science; 2016. 927 p.
